# A network analysis of depressive symptoms and metabolomics

**DOI:** 10.1017/S0033291723001009

**Published:** 2023-11

**Authors:** Arja O. Rydin, Yuri Milaneschi, Rick Quax, Jie Li, Jos A. Bosch, Robert A. Schoevers, Erik J. Giltay, Brenda W. J. H. Penninx, Femke Lamers

**Affiliations:** 1 Department of Psychiatry, Amsterdam UMC location Vrije Universiteit Amsterdam, Boelelaan 1117, Amsterdam, The Netherlands; 2 Amsterdam Public Health, Mental Health Program, Amsterdam, The Netherlands; 3 Computational Science Lab, Faculty of Science, Informatics Institute, University of Amsterdam, Amsterdam, The Netherlands; 4 Clinical Psychology, Faculty of Social and Behavioural Sciences, University of Amsterdam, Amsterdam, The Netherlands; 5 Department of Psychiatry, Faculty of Medical Sciences, University Medical Centre Groningen, University of Groningen, Groningen, The Netherlands; 6 Department of Psychiatry, Leiden University Medical Centre, Leiden University, Leiden, The Netherlands; 7 Department of Psychiatry and Neuroscience Campus Amsterdam, Amsterdam UMC, Vrije Universiteit, Amsterdam Public Health Research Institute, Amsterdam, The Netherlands

**Keywords:** Atypical depression, mixed graphical models, network analysis

## Abstract

**Background:**

Depression is associated with metabolic alterations including lipid dysregulation, whereby associations may vary across individual symptoms. Evaluating these associations using a network perspective yields a more complete insight than single outcome-single predictor models.

**Methods:**

We used data from the Netherlands Study of Depression and Anxiety (*N* = 2498) and leveraged networks capturing associations between 30 depressive symptoms (Inventory of Depressive Symptomatology) and 46 metabolites. Analyses involved 4 steps: creating a network with Mixed Graphical Models; calculating centrality measures; bootstrapping for stability testing; validating central, stable associations by extra covariate-adjustment; and validation using another data wave collected 6 years later.

**Results:**

The network yielded 28 symptom-metabolite associations. There were 15 highly-central variables (8 symptoms, 7 metabolites), and 3 stable links involving the symptoms Low energy (fatigue), and Hypersomnia. Specifically, fatigue showed consistent associations with higher mean diameter for VLDL particles and lower estimated degree of (fatty acid) unsaturation. These remained present after adjustment for lifestyle and health-related factors and using another data wave.

**Conclusions:**

The somatic symptoms Fatigue and Hypersomnia and cholesterol and fatty acid measures showed central, stable, and consistent relationships in our network. The present analyses showed how metabolic alterations are more consistently linked to specific symptom profiles.

## Introduction

Major Depressive Disorder (MDD) is a condition with a high burden of disease, which is partly due to comorbidity with other highly disabling somatic conditions such as cardiometabolic disorders (Gold et al., 2020; Liu et al., 2020; Otte et al., 2016). The metabolic alterations involved in cardiometabolic disorders may represent a common biological pathway connecting cardiometabolic disorders to MDD as well (Milaneschi, Lamers, Berk, & Penninx, 2020; Milaneschi, Simmons, van Rossum, & Penninx, 2019). Consistent with this hypothesis, metabolomics research using lipidomic panels identified associations between depression and higher levels of very low density lipoprotein (VLDL) and triglyceride particles, and negative associations with high density lipoprotein (HDL) (Bot et al., 2020). Furthermore, such metabolic signatures appear to be more consistently associated with the presence and severity of depression rather than anxiety (De Kluiver et al., 2021).

Depression is highly heterogeneous, and the link with metabolic alterations may vary as a function of the symptom profile expressed, with certain depressive symptoms being more strongly linked to metabolic alterations. Indeed, studies show that metabolic alterations (e.g. high visceral adipose tissue, inflammation, low HDL levels) are more strongly connected with atypical symptoms that characterise altered energy balance, such as Sleeping too much, Increased appetite, and Low energy levels (Alshehri et al., 2021; Beijers et al., 2019; Brydges et al., 2022; Lamers, Milaneschi, De Jonge, Giltay, & Penninx, 2018; Milaneschi et al., 2020).

The majority of studies investigating associations between depression and metabolic markers have examined mainly univariate associations (e.g. single outcome-single predictor models). Merely studying individual associations, however, does not do justice to the complexity and heterogeneous nature of depression. A growing field of research attempts to incorporate the heterogeneity of depression by applying network theory and models to these data (Borsboom, 2017; Borsboom et al., 2021; Borsboom & Cramer, 2013; Boschloo, van Borkulo, Borsboom, & Schoevers, 2016; Fried et al., 2017, 2020; Isvoranu et al., 2020; Van Borkulo et al., 2015). This approach has the advantage that it allows us to find associations in a system, conditioning on all variables simultaneously. Using this analytical approach, Kappelmann et al. (2021) studied links between 5 different polygenic scores of immune-metabolic traits and 7 depressive symptoms using a network model, finding that the C-Reactive Protein (CRP) polygenic score was associated with change in appetite, and to a lesser extent with anhedonia. Other studies found associations between CRP, Tumour Necrosis Factor *α* (TNF-*α*), and Interleukin-6 (IL-6) with depressive symptoms of Sleep problems and Appetite/Weight changes (Fried et al., 2020; Moriarity & Alloy, 2020; Moriarity, Horn, Kautz, Haslbeck, & Alloy, 2021). Whereas these networks capture a bigger picture of associations between psychopathological symptoms and (small sets of) biomarkers, a network-wide approach has yet to be applied to large panels of metabolic markers (i.e. metabolomics).

To close above gaps, this paper explored associations between depressive symptoms and a metabolomic panel of 46 markers, using Mixed Graphical Models to create a network with these variables. Lipid-based metabolites and individual depressive symptoms are expected to be highly intra- and inter-correlated; to disentangle the broad correlation matrix we applied network centrality measures allowing to identify the most relevant metabolite-symptom links in the network. One-time only assessment may not be representative of long lasting underlying connections between metabolites and depressive symptoms, because such measures could vary over time and be influenced by several factors such as medication, smoking, and BMI. Furthermore, these types of networks are prone to overfitting. Therefore, in order to identify the more reliable and consistent symptom-metabolite links we conducted a stability analysis of the edges in the network and we validated the most stable connections using similar data collected from the same subjects 6 years later, additionally adjusting for different sets of covariates.

## Methods

### Sample

Data are from participants from the Netherlands Study for Depression and Anxiety (NESDA), an ongoing longitudinal cohort study on the long-term course and consequences of depressive and anxiety disorders. Penninx et al. (2008) and Penninx et al. (2021) provide a description of the study rationale, design, and methods. At baseline, 2981 participants were included between the ages of 18 and 65, who were recruited between 2004 and 2007 from the community (19.0%), primary care (54.0%), and specialised mental health care setting (27.0%). The 6-year follow-up (age range 23–72) consisted of 2256 participants.

Participants were either healthy controls or had a current or prior history of depressive and/or anxiety disorder. Diagnoses of depression and anxiety disorders were determined with the use of the Composite Interview Diagnostic Instrument (CIDI) – lifetime version 2.1, which assesses diagnostic criteria of the Diagnostic and Statistical Manual of Mental Disorders (DSM)-IV. Participants were excluded when they did not speak fluent Dutch, or when they had a primary clinically overt other psychiatric diagnosis, such as bipolar, psychotic, obsessive compulsive, or severe addictive disorder. The Ethical Committee of all universities participating approved the NESDA project, and all participants provided written informed consent. Data collection included an extensive interview and blood collection.

The network model required complete data on all measures; thus, we selected 2498 participants with complete baseline data on all metabolites and depressive symptoms for the main analyses. For 1745 of these participants complete data was available at 6-year follow-up.

### Depressive symptoms

Depressive symptoms (in the week before the baseline interview and the 6-year follow-up interview) were measured using the 30-item self-report Inventory of Depressive Symptomatology (IDS-SR30) (Rush, Gullion, Basco, Jarrett, & Trivedi, 1996). Each symptom is represented by 4 statements representing an ordinal scale in the range 0–3 (e.g. 0 = ‘I do not feel sad’, 3 = ‘I feel sad nearly all the time’). For interpretation, symptoms were divided into 2 groups (online Supplementary Table S1): 13 somatic (e.g. Aches and pains, Sleeping too much) and 16 mood/cognition (e.g. Feeling sad, General interest) symptoms. This grouping is based on a factor analysis performed by Wardenaar et al. (2010), where the symptoms were clustered into 3 factors (i.e. ‘mood/cognition’, ‘anxiety/arousal’, and ‘sleep’). As previously done by Vreijling et al. (2021), the ‘anxiety/arousal’ and ‘sleep’ factors were merged into a new factor labelled ‘somatic’, as the sleep profile contained only 4 variables. The symptom Diurnal variation of mood was removed from either category, as it associates poorly with both groups (Rush et al., 1996; Vreijling et al., 2021; Wardenaar et al., 2010). The symptoms Weight change and Appetite change were split into 4 separate variables: Weight or Appetite increase and decrease.

### Metabolomic biomarkers measurement

Participants were asked to provide overnight fasted blood samples during the baseline interview in the morning (between 08.30 and 09.30). Plasma samples were stored in the ethylenediaminetetraacetic acid (EDTA) detergent, and kept at a temperature of −80 °C until they were assayed. The baseline samples were shipped in 2 batches (April and December 2014, referred to as batch 1 and batch 2 respectively); the 6-year follow-up samples were shipped in a single batch. Assessments were based on a proton nuclear magnetic resonance (NMR) platform (Nightingale Health Ltd., Helsinki, Finland) (Soininen, Kangas, Würtz, Suna, & Ala-Korpela, 2015) quantified concentrations of several lipid-related metabolites and their ratios.

Among the measures provided by the platform, we selected concentrations of 51 lipids, fatty acids, and low molecular weight metabolites as in a previous study by De Kluiver et al. (2021). This study also found 13 metabolites to be batch-sensitive, meaning the metabolite levels were different across batches; we adjusted for batch. Additional sub-measures and ratios of these lipoproteins (i.e. lipid composition and particle concentration measures of lipoprotein subclasses and lipid and fatty acids ratios) were not included in the current analyses because of redundancy. Furthermore, when more than 2.5% of the participants had a missing for the same metabolite, that metabolite was removed from the dataset. This resulted in the removal of 5 metabolites (columns). Subsequently, there were 79 subjects (rows) with missing values (2.7% of the total number of subjects); these subjects were removed from the dataset. Applying the above selection criteria resulted in 46 metabolites for final analysis. These metabolites were classified into 11 subcategories (online Supplementary Table S1) according to the classification provided by Soininen et al. (2015).

Data were processed according to the manufacturer's standardised protocol previously applied in other large scale studies (Bot et al., 2020). A value of 1 was added to each value, and the natural log transformation was applied. Values that deviated >5 s.d.s from the mean were set as missing.

### Descriptive variables and covariates

Baseline sex, age and sample shipment (batch 1 and 2) were used to adjust metabolite levels for the network analysis, For the regression analyses in the validation step, different sets of covariates were used: sex, age, statin use (Anatomic Therapeutic chemical C10AA, abbreviation ATC), antidepressant (AD) use (tricyclic, ATC N06AA; selective serotonin reuptake inhibitors, ATC N06AB; and other antidepressants, N06AF), Body Mass Index (BMI) based on measured height and weight, and current smoking status (yes/no). Medication info was obtained based on container inspection during the interview. Similar variables were available at the 6-year follow-up.

### Statistical analyses

Analyses were conducted in R (v 3.6.1; R foundation for Statistical Computing, Vienna, Austria, 2016. URL: https://www.R-project.org/). The ‘mgm’ (version 1.2–12), ‘qgraph’ (version 1.6.9), ‘Rmisc’ (version 1.5.1) packages for the R statistical software were used. Networks were visualised with the *qgraph*-package (Epskamp, Cramer, Waldorp, Schmittmann, & Borsboom, 2012). Variables were reported as a percentage, or mean ± standard deviation as appropriate. Metabolite concentrations were first regressed on sex, age and batch number using ordinary least squares regression; the residuals were saved and used a model input.

The analyses involved 4 steps: creating the network with MGM in the baseline data; calculating 5 network centrality measures for all variables in the network; testing the links for stability; and lastly performing a linear regression on the stable links connected to central nodes, additionally correcting for sets of covariates and replicating in the 6-year follow-up dataset. We elaborate on these 4 steps below.

#### Mixed graphical models

Depressive symptoms (categorical) and residualised metabolite values (Gaussian) were fed into the model using the MGM package (Haslbeck & Waldorp, 2015) (see online Supplementary Fig. S2 for distributions). Each variable is treated as a random variable, and the distribution of the whole set of variables is assumed to be factorised according to an undirected graph *G.* The connection of a (multivariate) probability distribution and a graph is formalised by the Global Markov Property. The variables (represented as nodes) are connected through conditional distributions. These distributions are part of the exponential family. The parameters belonging to this family function as the edge weights of the network. Some elaboration of the mathematics is provided in the online Supplemental materials.

We did not apply cross-validation nor an EBIC parameter, which are used as a penalty parameter to prohibit overfitting; the associations between symptoms and metabolites were likely to be weaker than within-group associations; by applying a penalty we would not have found these more subtle associations. The nodes were grouped together a clustering function within the package.

#### Network centrality measures

The network visualisation provides an intuitive overview of how variables (nodes) are connected with one another, although the interpretation of the associations (edges) is not directly straight-forward. Therefore, to add to the visual inspection, 5 different network measures were used. These capture different properties of the network nodes. The 5 measures were scaled between 0 and 1, and ranked individually. Mathematical definitions can be found in Rodrigues (2019).

***Betweenness*** *–* Betweenness counts how often a node is in the shortest path between all other node pairs. If a node often appears on the shortest path, it may have a mediating function for many node sets.

***Closeness*** *–* Closeness for a node is the inverted sum of its average distance (according to some predefined metric) to all other nodes. If a node has a high closeness it is very central in the network, which is an indication of being able to spread ‘information’ efficiently through a network.

***Weighted degree*** – The weighted degree sums the weight of all edges connected to a node, and informs us about the connectedness to other nodes. Thicker edges mean stronger associations If a node has many small incoming edges, it might have a similar weighted degree as a node with one big edge. The measure can help identify hubs.

***Ratio betweenness and degree*** – If a node has a high betweenness, but also a high weighted degree, then the ratio of the two measures is low, and that node is relatively *node-stable*. In contrast if a node has high betweenness, but *low* degree, the ratio is high: perturbation in the system regarding that area of the network can have a big effect on the system. Consequently, nodes with high betweenness/degree ratio could be interesting as target for an intervention. This is under the assumption that overall degree scales proportionally with in-degree.

***Ratio closeness and degree*** – Similar to how betweenness and degree can say something about node-stability, closeness and degree can do the same thing. A high ratio means this node is less stable, and therefore could be interesting in terms of having a big effect on the system when the node changes (again under the assumption that in-degree and total degree are positively correlated). The links in these networks are correlations, and although correlation does not imply causation (the links do not need to be causal), causation does imply correlation; in this network, causal connections could be forming a subnetwork. These 2 ratio measures have potential to capture some of the causal connections.

#### Stability analysis

Stability analyses were performed on all potential edges in the network through bootstrapping (Haslbeck & Waldorp, 2015). This method runs the model 50 times for potential edges, and resulted in an *edge-stability* assessment for 2850 links^†^^1^. We selected edges which were connected to variables scoring highest on at least 1 of the 5 network measures. For these edges we showed the 95% confidence interval (2.5th and 97.5th percentiles) and mean of the edge strengths. Additionally, we provided the fraction of times that the edge was present in the bootstraps. This provides an edge-stability measure for the links that were found to be important in the centrality assessments.

#### Validation

As a validation step for the stable links connected to central nodes, we performed a linear regression. Because these links might arise from unknown mechanisms, we corrected for different sets of covariates: no additional covariates (only sex and age); statin use; AD use; BMI; BMI and smoking; BMI, smoking and AD use; and BMI, smoking AD and statin use. Lastly, we compared these associations with the 6-year follow-up dataset as an additional step to confirm consistency in the findings.

## Results

### Descriptives

The baseline sample included 2498 subjects with complete data on metabolites and depressive symptoms; the 6-year follow-up sample included 1745. The descriptives and covariates are provided in Table 1. Properties of the datasets after cleaning were similar to the original datasets, apart from sample size, which was reduced by 16% in the baseline, and 23% in the 6-year follow-up datasets.

**Table 1.** Descriptives and covariates of the NESDA baseline and six-year follow-up datasets

	Baseline dataset	Six-year follow-up
N	2498	1745
Sex (% female)	66	66
Mean age (years, std)	42 ± 13	48 ± 13
MDD diagnosis (%)	36.3	15
Smoking (%)	37	27
BMI ± std	26 ± 4.9	25 ± 7.0
Tricyclic antidepressants (%)	2.8	2.9
Selective serotonin reuptake inhibitors (%)	17.4	11.4
other antidepressants (%)	5.6	4.9
Statins (%)	7.1	9.7

Online Supplementary Fig. S1 shows the distribution of the 30 IDS-SR items at baseline. The symptoms are ranked from most prevalent to least prevalent. The most endorsed symptoms included Problems sleeping during the night, Leaden paralysis, and Problems falling asleep. The distribution of the 46 metabolites can be found in online Supplementary Fig. S2.

### Network analyses

**Figure 1.**
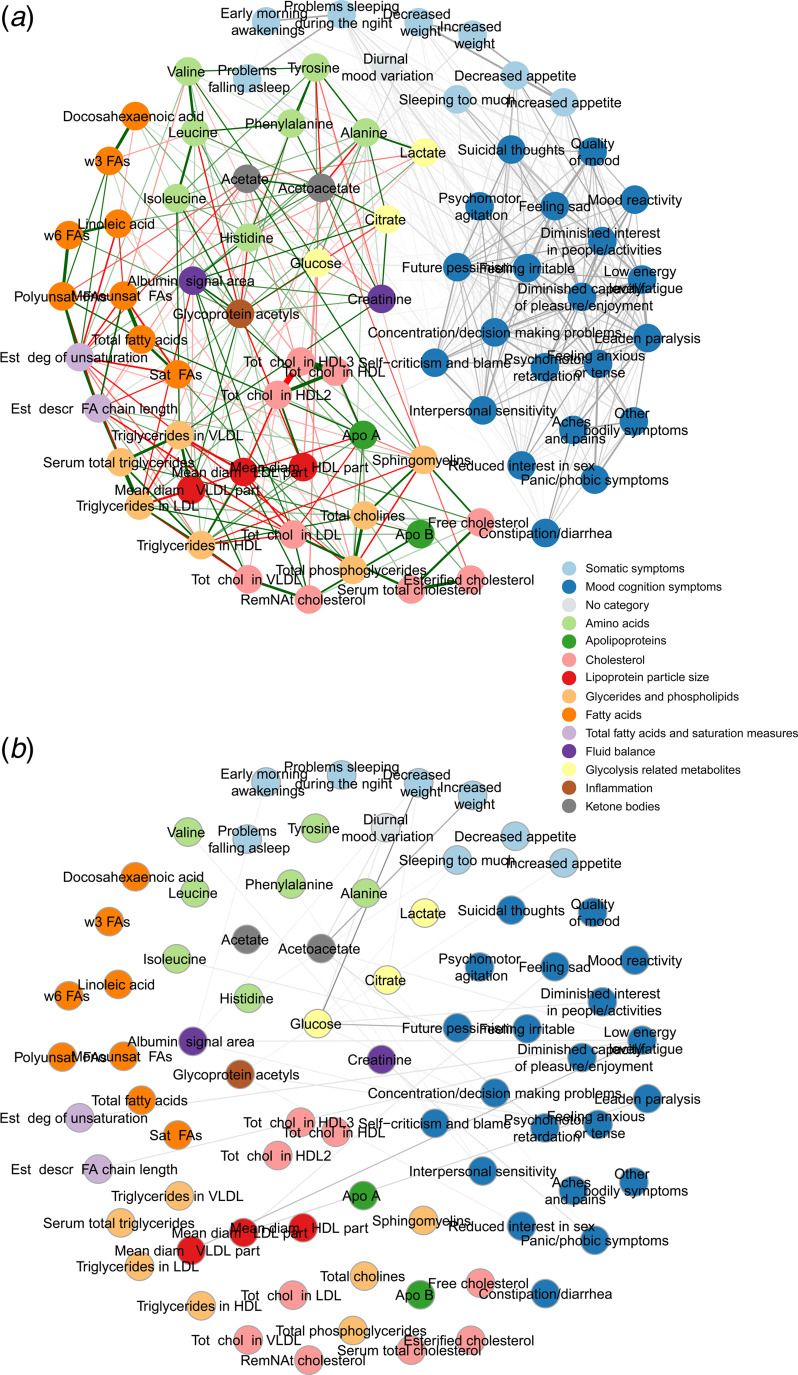
MGM network visualisation of pairwise associations between depressive symptoms and metabolites.

Figure 1a show the MGM network of depressive symptoms and metabolites (see online Supplementary Table S1 for extended legend). As expected, the MGM shows a clustering of symptoms, and a clustering of metabolites. A total of 28 bridge associations (associations between a metabolite and a depressive symptom) were visible. These bridge associations are highlighted in Fig. 1b by leaving out all intra-group associations. Figure 1b shows 8 somatic symptoms (61.5% of all somatic symptoms) and 8 mood/cognition symptoms (50.0% of all mood/cognition symptoms) are connected to metabolites.

Several nodes had 5 or more bridge associations: glucose (variable 72), connected to 6 symptom variables; acetoacetate (variable 76) had 5 connections with symptom variables; Low energy levels/fatigue (16) was connected to 5 metabolites.

There are no direct connections of symptoms with apolipoproteins (dark-green) cholesterol (pink), glycerides and phospholipids (light-orange) and fatty acids (dark-orange). However, connections are visible between depressive symptoms and amino acids (light-green); lipoprotein particle size (red); total fatty acids and saturation measures (light-purple); fluid balance (dark-purple); glycolysis related metabolites (yellow); inflammation (brown) and lastly, ketone bodies (light-blue).

### Network centrality measures

**Figure 2.**
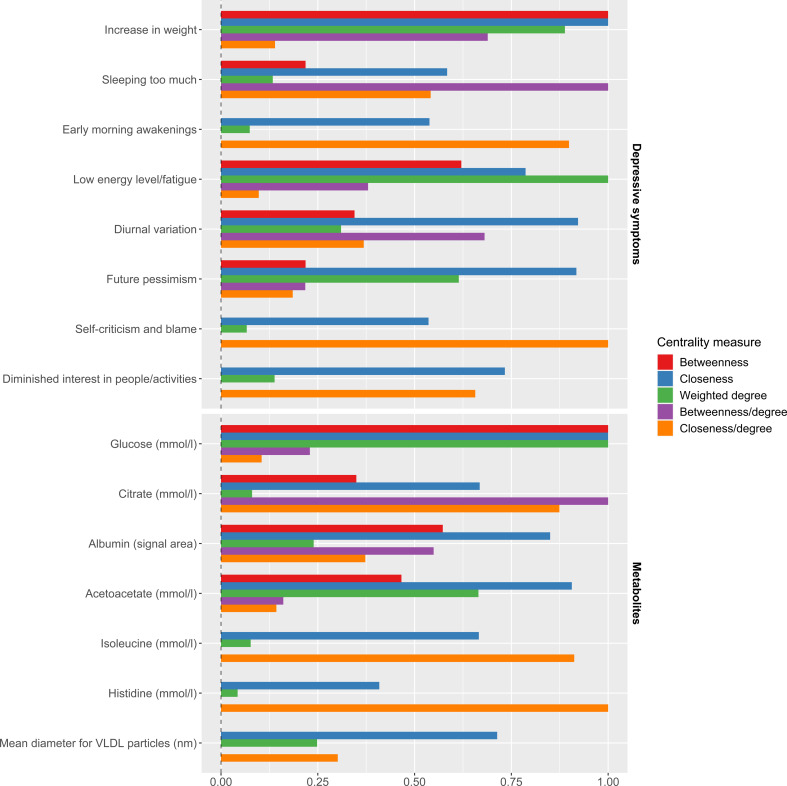
Variables (depressive symptoms and metabolites) which are ranked in the top-3 for at least one of the 5 centrality measures.

Mere visualisation of number of connections alone might miss which variables have high centrality measures, or have a high connectedness to the other nodes in the network. Figure 2 shows the variables that score high on the 5 network measures, that is those that are among the top 3 for at least 1 of the 5 centrality measures (8 depressive symptoms of which 3 somatic, and 7 metabolites). The measure values have been scaled for comparability.

Increase in weight, Sleeping too much, and Low energy levels stand out for the depressive symptoms. For the metabolites we see glucose has high centrality measures, histidine has the highest score for closeness/degree, and mean diameter size for VLDL particles has a high closeness value. In the next step we show that some of these variables also had a high stability.

### Stability analysis

For the variables with a high centrality score we evaluated the edge-stability, an important measure when evaluating associations in a network. Bootstraps were performed on the node pairs of which at least one had the highest score for one of the five network measures, including: Sleeping too much; Low energy level/fatigue; Self-criticism and blame; glucose, citrate; and histidine. The model was bootstrapped 50 times for each potential link.

**Figure 3.**
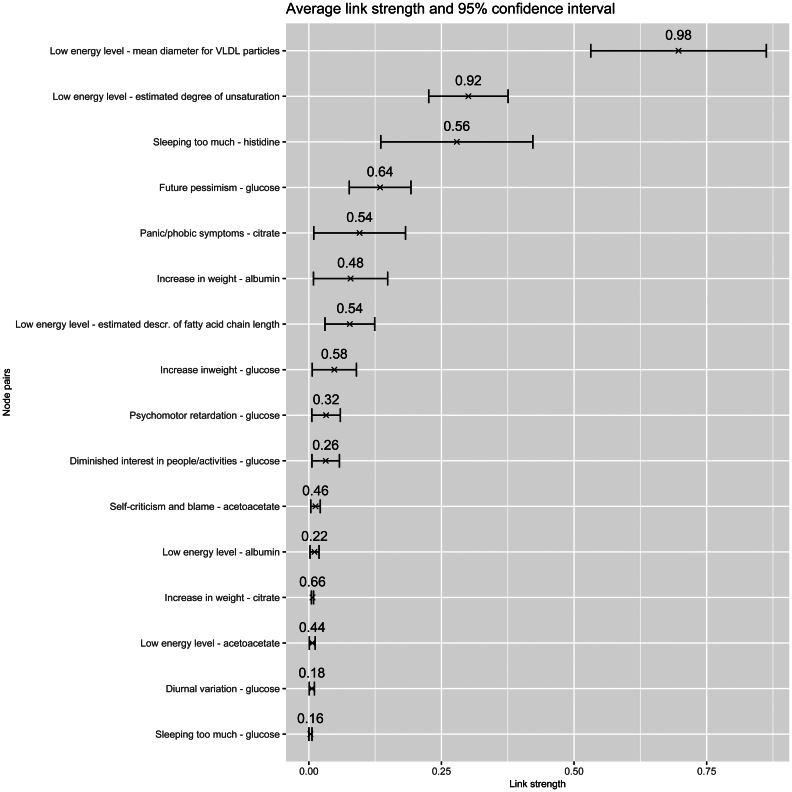
Stability analysis results using a bootstrapping method, applied to links that connected to variables that scored highest on at least 1 out of 5 centrality measures.

In Fig. 3, the bars show the average edge-strength for each node pair and the 95% confidence interval. The numbers in the graph stand for the fraction of how often an edge was present in one of the 50 bootstraps. The edges are ranked on average edge-strength. What stands out in Fig. 5 are the top two links, Low energy level and mean diameter of VLDL particles (16–49), and Low energy level and Estimated degree of unsaturation (16–68): these have an edge-stability of 98% and 92%, respectively. The third in rank, Sleeping too much and histidine (4–32), also has a relatively high and stable association.

### Validation through regression baseline and 6-year follow-up

Lastly, we performed a validation step. We estimated the 3 most stable associations through a linear regression adjusting for different sets of covariates on baseline dataset. As a consistency check the regressions were performed using 6-year follow-up data.

The linear regressions are the last step of the statistical analyses, done to check what the influence of covariates was on the associations found in the network and stability analysis, and to check whether the association is consistent over time. As shown in Table 2, there was a significant association for all three pairs in the baseline dataset: Low energy level was positively associated with mean diameter of VLDL particles and negatively with estimated degree of unsaturation; Sleeping too much was negatively associated with histidine. The associations of Low energy level with mean diameter of VLDL particles and estimated degree of unsaturation were confirmed in the 6-year follow-up data. Overall, adjustment for lifestyle and medications partially reduced the strength of the associations, indicating a role of these factors in the link between depressive symptoms and metabolites.

**Table 2.** Results of the linear regression

	Extra corrected for	T0		FU6	
Var names	Sex, age, and	beta	*p*	beta	*p*
Low energy level – Mean diameter of VLDL particles		0.0055 ± 0.0029	**<0**.**001**	0.013 ± 0.0048	**<0**.**001**
	AD use	0.0046 ± 0.0029	**<0**.**01**	0.012 ± 0.0049	**<0**.**001**
	Statin use	0.0053 ± 0.0029	**<0**.**001**	0.012 ± 0.0048	**<0**.**001**
	BMI	0.0035 ± 0.0027	**0**.**012**	0.011 ± 0.0048	**<0**.**001**
	BMI, smoking	0.0024 ± 0.0027	0.083	0.010 ± 0.0048	**<0**.**001**
	BMI, smoking, AD use	0.002 ± 0.0028	0.16	0.0094 ± 0.0048	**<0**.**001**
	BMI, smoking, AD, statin	0.0020 ± 0.0028	0.16	0.0089 ± 0.0048	**<0**.**001**
Low energy level – Estimated degree of unsaturation		−0.0052 ± 0.0030	**<0**.**001**	−0.0082 ± 0.0038	**<0**.**001**
	AD use	−0.0043 ± 0.0030	**0**.**0055**	−0.0069 ± 0.0037	**<0**.**001**
	Statin use	−0.0053 ± 0.0030	**<0**.**001**	−0.0084 ± 0.0038	**<0**.**001**
	BMI	−0.0042 ± 0.0029	**<0**.**01**	−0.0070 ± 0.0036	**<0**.**001**
	BMI, smoking	−0.0029 ± 0.0029	**<0**.**01**	0.0064 ± 0.0036	**<0**.**001**
	BMI, smoking, AD use	−0.0023 ± 0.0030	0.13	0.0053 ± 0.0037	**<0**.**01**
	BMI, smoking, AD, statin	−0.0023 ± 0.0030	0.13	0.0054 ± 0.0037	**<0**.**01**
Sleeping too much – Histidine		−0.0016 ± 0.0013	**0**.**022**	−0.0012 ± 0.0020	0.25
	AD use	−0.0014 ± 0.0014	**0**.**044**	−0.00093 ± 0.0020	0.36
	statin use	−0.0016 ± 0.0013	**0**.**021**	−0.0011 ± 0.0020	0.28
	BMI	−0.0015 ± 0.0013	**0**.**03**	−0.0010 ± 0.0020	0.32
	BMI, smoking	−0.0014 ± 0.0013	**0**.**037**	−0.00091 ± 0.0020	0.37
	BMI, smoking, AD use	−0.0013 ± 0.0014	0.06	−0.00076 ± 0.0020	0.45
	BMI, smoking, AD, statin	−0.0013 ± 0.0014	0.054	−0.00071 ± 0.0020	0.49

Significant *p* values are in boldface.

## Discussion

In light of high associations between depression and cardiovascular disease risk, this study aimed to examine co-expression of metabolic markers and depressive symptoms using network modelling. The network contained a 46 parameter lipidomics panel and 30 depressive symptoms. Analyses showed patterning of associations across symptoms and metabolites whereby some depressive symptoms showed a relatively more central role (e.g. Low energy level, Sleeping too much and Weight increase). The connections between these symptoms and specific metabolites were stable, and consistent across two different time assessments, most notably associations between Low energy level and VLDL particle size and fatty acid saturation. These associations replicate prior findings (Brydges et al., 2022; Drevets, Wittenberg, Bullmore, & Manji, 2022; Grela et al., 2016; Knowles et al., 2017; Lamers et al., 2018; Milaneschi et al., 2020). VLDL particle size summarises the size of cholesterols and is also highly correlated with several cholesterol variables (Bowden, Wilson, & Beaujean, 2011; Sacks & Campos, 2003).

Depressive symptoms such as Sleeping too much, Low energy level and Increase in weight form a subgroup referred to as atypical, energy-related symptoms. These share associations with cardiometabolic diseases (Alshehri et al., 2021; Lamers et al., 2018), and inflammatory and metabolic dysregulations (Brydges et al., 2022; Lamers et al., 2020; Milaneschi et al., 2020), which is congruent with the present findings. This clustering of atypical energy-related symptoms sharing associations with heightened levels of inflammation is referred to as Immunometabolic Depression (IMD).

Network centrality measures can give an interpretation of the potential role of a node in the network. Low energy level scored high on weighted degree, implying the node is a stable factor in the network. Furthermore, the node scored high on closeness and betweenness, which could mean Low energy might have a mediating role in its spreading effects to other variables. Mean diameter for VLDL particles showed mostly a high score for closeness, which is a measure that indicates the spread of information through a system.

Sleeping too much scored high on the two ratio centrality measures, implying this variable might have potential to alter the system. Lastly, histidine scored highest on the ratio measure closeness/degree, which suggests that histidine can spread information efficiently and is node-unstable, meaning it can perturb the system when changed. Histidine is a semi-essential amino acid involved in protein interactions and synthesis (Brosnan & Brosnan, 2020; Liao, Du, Meng, Pang, & Huang, 2013). Lowered histidine levels and sleep deprivation are correlated (Nakamura et al., 2022; Sasahara, Fujimura, Nozawa, Furuhata, & Sato, 2015; Thalacker-Mercer & Gheller, 2020).

We saw a decrease in associations strengths mainly after correcting for BMI. This trend was most pronounced in the association between Low energy level and mean diameter for VLDL particles, but also visible in the association between Low energy level and degree of unsaturation (of fatty acids). Both VLDL and fatty acids share associations with BMI (Arner & Rydén, 2015; Björnson, Adiels, Taskinen, & Borén, 2017; Boden, 2008; Karpe, Dickmann, & Frayn, 2011; Lewis, Uffelman, Szeto, & Steiner, 1993; Mittendorfer, Patterson, & Klein, 2003; Mittendorfer, Yoshino, Patterson, & Klein, 2016; Nielsen & Karpe, 2012), indicating that BMI might be on the causal pathway. However, BMI did not fully explain the whole association, and other mechanisms could carry responsibility for the connection. For example, fatty acids are a key component of energy production in mitochondria, and mitochondrial dysfunction is linked to depression (Milaneschi et al., 2022). Another potential explanation of the association between lipids and depression (and specifically fatigue) could be their shared association with inflammation (Glass & Olefsky, 2012; Karshikoff, Sundelin, & Lasselin, 2017; Lee & Giuliani, 2019; Lin, Huang, & Su, 2010; Louati & Berenbaum, 2015; Masoodi, Kuda, Rossmeisl, Flachs, & Kopecky, 2015; Van Diepen, Berbée, Havekes, & Rensen, 2013; Zhang et al., 2018; Zhong et al., 2019).

It is generally known that biomarkers are not independent of each other, and neither are depressive symptoms; applying a network to the data respects these underlying distributions and interdependencies of the variables. Furthermore, we did not only show the resulting networks (which are prone to overfitting), but also performed a stability test on all the links that had a high centrality. This is an important step in the network analysis as spurious links should be filtered out. The validation confirms that the associations did not merely arise through confounding factors. NESDA does not only provide a large dataset, it also allowed us to check for consistency using the 6-year follow-up, which significantly strengthens the legitimacy of the found associations. Networks can better capture which variables are associated, as depression is known to be a highly heterogeneous disorder. Lastly, this approach allows for more layers of disease outcomes or biomarkers to be added.

Some critical remarks are warranted. While the centrality measures are intuitive, and the implementation is straight-forward; more specialised bridging-measures could be applied in future work. Examples are Jensen et al. (2016) and Valente and Fujimoto (2010). These are more complex to implement but give a more fine-tuned interpretation of the network's nodes. We also did not make use of a penalisation parameter, which is applied to prohibit the finding of spurious associations in the network. We did this because we were interested in the associations between symptoms and metabolites, which are substantially weaker than symptom-symptom and metabolite-metabolite associations. Penalising all the links similarly would have resulted close to no symptom-metabolite links. However, we ensured elimination of potential spurious results through the stability analysis and validation steps. When analysing networks with either only continuous, or only discrete random variables, we recommend the Network Comparison Test by Van Borkulo et al. (2022). This is a useful tool to test for stability; however, our data was mixed, so this package could not be applied to this network. Lastly, the methodology is new, but associations between depressive symptoms and metabolites have been studied before. The added benefit would be to also add extra layers of biomarkers or disease outcomes. An obvious limitation of studying cross-sectional data is that causality is not inferable. This work offers preliminary insights in the associations between metabolites and depressive symptoms, and invites further investigations, e.g., longitudinal analyses based on the findings of this paper.

We illustrate that applying the network approach to biological and symptom data, provides useful insights in core aspects of the biology-symptom links. Some symptoms are more linked to certain metabolites than others, and serve as bridge-associations between the symptoms-group and metabolite-group. This should be used in future large-scale biological and psychiatric datasets. Lastly, our findings suggest studying metabolic dysregulations in a subgroup of patients suffering from atypical, energy-related symptoms.

Due to the complexity of the interplay of symptoms and metabolites, and especially the differential expression of depressive symptoms, the application of adequate statistical techniques is essential. By creating a network of individual depressive symptoms and a full lipidomic panel we found a subset of symptoms and markers showing strong connections. Our results thereby add to the needed insights of MDD's heterogeneity and illustrate the important roles of Low energy level, VLDL cholesterol particle size, and saturation of fatty acids.

## Supporting information

Rydin et al. supplementary material 1 Rydin et al. supplementary material

Rydin et al. supplementary material 2 Rydin et al. supplementary material

Rydin et al. supplementary material 3 Rydin et al. supplementary material

Rydin et al. supplementary material 4 Rydin et al. supplementary material

## Footnotes

† The notes appear after the main text.

## Note

1 The maximum number of edges in an undirected graph with *n* nodes equals *n × (n-1) ÷ 2.* In this case, *n = 76,* and 76 × 75 ÷ 2 = 2850.

## Supplementary material

The supplementary material for this article can be found at https://doi.org/10.1017/S0033291723001009

## References

[ref1] Alshehri, T., Mook-Kanamori, D. O., van Dijk, K. W., Dinga, R., Penninx, B. W., Rosendaal, F. R., … Milaneschi, Y. (2021). Metabolomics dissection of depression heterogeneity and related cardiometabolic risk. Psychological Medicine, 53(1), 1–10. 34078486 10.1017/S0033291721001471 PMC9874986

[ref2] Arner, P., & Rydén, M. (2015). Fatty acids, obesity and insulin resistance. Obesity Facts, 8(2), 147–155. 25895754 10.1159/000381224 PMC5644864

[ref3] Beijers, L., Wardenaar, K. J., Bosker, F. J., Lamers, F., van Grootheest, G., de Boer, M. K., … Schoevers, R. A. (2019). Biomarker-based subtyping of depression and anxiety disorders using Latent Class Analysis. A NESDA study. Psychological Medicine, 49(4), 617–627. 29860945 10.1017/S0033291718001307 PMC6393228

[ref4] Björnson, E., Adiels, M., Taskinen, M.-R., & Borén, J. (2017). Kinetics of plasma triglycerides in abdominal obesity. Current Opinion in Lipidology, 28(1), 11–18. 27898581 10.1097/MOL.0000000000000375

[ref5] Boden, G. (2008). Obesity and free fatty acids. Endocrinology and Metabolism Clinics of North America, 37(3), 635–646. 18775356 10.1016/j.ecl.2008.06.007 PMC2596919

[ref6] Borsboom, D. (2017). A network theory of mental disorders. World Psychiatry, 16(1), 5–13. 28127906 10.1002/wps.20375 PMC5269502

[ref7] Borsboom, D., & Cramer, A. O. (2013). Network analysis: An integrative approach to the structure of psychopathology. Annual Review of Clinical Psychology, 9, 91–121. 10.1146/annurev-clinpsy-050212-185608 23537483

[ref8] Borsboom, D., Deserno, M. K., Rhemtulla, M., Epskamp, S., Fried, E. I., McNally, R. J., … Costantini, G. (2021). Network analysis of multivariate data in psychological science. Nature Reviews Methods Primers, 1(1), 1–18.

[ref9] Boschloo, L., van Borkulo, C. D., Borsboom, D., & Schoevers, R. A. (2016). A prospective study on how symptoms in a network predict the onset of depression. Psychotherapy and Psychosomatics, 85(3), 183–184. 27043457 10.1159/000442001

[ref10] Bot, M., Milaneschi, Y., Al-Shehri, T., Amin, N., Garmaeva, S., Onderwater, G. L., … Vogelzangs, N. (2020). Metabolomics profile in depression: A pooled analysis of 230 metabolic markers in 5283 cases with depression and 10145 controls. Biological Psychiatry, 87(5), 409–418. 31635762 10.1016/j.biopsych.2019.08.016 PMC11921392

[ref11] Bowden, R. G., Wilson, R. L., & Beaujean, A. A. (2011). LDL particle size and number compared with LDL cholesterol and risk categorization in end-stage renal disease patients. Journal of Nephrology, 24(6), 771. 21360474 10.5301/JN.2011.6376 PMC4313745

[ref12] Brosnan, M. E., & Brosnan, J. T. (2020). Histidine metabolism and function. The Journal of Nutrition, 150(Supplement_1), 2570S–2575S. 33000155 10.1093/jn/nxaa079 PMC7527268

[ref13] Brydges, C. R., Bhattacharyya, S., Dehkordi, S. M., Milaneschi, Y., Penninx, B., Jansen, R., … Kastenmüller, G. (2022). Metabolomic and inflammatory signatures of symptom dimensions in major depression. Brain, Behavior, and Immunity, 102, 42–52. 35131442 10.1016/j.bbi.2022.02.003 PMC9241382

[ref14] De Kluiver, H., Jansen, R., Milaneschi, Y., Bot, M., Giltay, E. J., Schoevers, R., & Penninx, B. W. (2021). Metabolomic profiles discriminating anxiety from depression. Acta Psychiatrica Scandinavica, 144(2), 178–193. 33914921 10.1111/acps.13310 PMC8361773

[ref15] Drevets, W. C., Wittenberg, G. M., Bullmore, E. T., & Manji, H. K. (2022). Immune targets for therapeutic development in depression: Towards precision medicine. Nature Reviews Drug Discovery, 21(3), 224–244. 35039676 10.1038/s41573-021-00368-1 PMC8763135

[ref16] Epskamp, S., Cramer, A. O., Waldorp, L. J., Schmittmann, V. D., & Borsboom, D. (2012). Qgraph: Network visualizations of relationships in psychometric data. Journal of Statistical Software, 48, 1–18.

[ref17] Fried, E. I., van Borkulo, C. D., Cramer, A. O., Boschloo, L., Schoevers, R. A., & Borsboom, D. (2017). Mental disorders as networks of problems: A review of recent insights. Social Psychiatry and Psychiatric Epidemiology, 52(1), 1–10. 27921134 10.1007/s00127-016-1319-z PMC5226976

[ref18] Fried, E. I., Von Stockert, S., Haslbeck, J., Lamers, F., Schoevers, R., & Penninx, B. (2020). Using network analysis to examine links between individual depressive symptoms, inflammatory markers, and covariates. Psychological Medicine, 50(16), 2682–2690. 31615595 10.1017/S0033291719002770

[ref19] Glass, C. K., & Olefsky, J. M. (2012). Inflammation and lipid signaling in the etiology of insulin resistance. Cell Metabolism, 15(5), 635–645. 22560216 10.1016/j.cmet.2012.04.001 PMC4156155

[ref20] Gold, S. M., Köhler-Forsberg, O., Moss-Morris, R., Mehnert, A., Miranda, J. J., Bullinger, M., … Otte, C. (2020). Comorbid depression in medical diseases. Nature Reviews Disease Primers, 6(1), 1–22. 10.1038/s41572-020-0200-2 32820163

[ref21] Grela, A., Rachel, W., Cole, M., Zyss, T., Zięba, A., & Piekoszewski, W. (2016). Application of fatty acid and lipid measurements in neuropsychiatry. Clinical Chemistry and Laboratory Medicine *(*CCLM*)*, 54(2), 197–206. 26351933 10.1515/cclm-2015-0394

[ref22] Haslbeck, J., & Waldorp, L. J. (2015). mgm: Estimating time-varying mixed graphical models in high-dimensional data. *arXiv preprint arXiv*:1510.06871.

[ref23] Isvoranu, A.-M., Guloksuz, S., Epskamp, S., van Os, J., Borsboom, D., & Investigators, G. (2020). Toward incorporating genetic risk scores into symptom networks of psychosis. Psychological Medicine, 50(4), 636–643. 30867074 10.1017/S003329171900045X PMC7093319

[ref24] Jensen, P., Morini, M., Karsai, M., Venturini, T., Vespignani, A., Jacomy, M., … Fleury, E. (2016). Detecting global bridges in networks. Journal of Complex Networks, 4(3), 319–329.

[ref25] Kappelmann, N., Czamara, D., Rost, N., Moser, S., Schmoll, V., Trastulla, L., … Khandaker, G. M. (2021). Polygenic risk for immuno-metabolic markers and specific depressive symptoms: A multi-sample network analysis study. Brain, Behavior, and Immunity, 95, 256–268. 33794315 10.1016/j.bbi.2021.03.024

[ref26] Karpe, F., Dickmann, J. R., & Frayn, K. N. (2011). Fatty acids, obesity, and insulin resistance: Time for a reevaluation. Diabetes, 60(10), 2441–2449. 21948998 10.2337/db11-0425 PMC3178283

[ref27] Karshikoff, B., Sundelin, T., & Lasselin, J. (2017). Role of inflammation in human fatigue: Relevance of multidimensional assessments and potential neuronal mechanisms. Frontiers in Immunology, 8, 21. 28163706 10.3389/fimmu.2017.00021 PMC5247454

[ref28] Knowles, E. E., Huynh, K., Meikle, P. J., Göring, H., Olvera, R. L., Mathias, S. R., … Curran, J. E. (2017). The lipidome in major depressive disorder: Shared genetic influence for ether-phosphatidylcholines, a plasma-based phenotype related to inflammation, and disease risk. European Psychiatry, 43, 44–50. 28365467 10.1016/j.eurpsy.2017.02.479 PMC5507360

[ref29] Lamers, F., Milaneschi, Y., De Jonge, P., Giltay, E. J., & Penninx, B. W. (2018). Metabolic and inflammatory markers: Associations with individual depressive symptoms. Psychological Medicine, 48(7), 1102–1110. 28889804 10.1017/S0033291717002483

[ref30] Lamers, F., Milaneschi, Y., Vinkers, C. H., Schoevers, R. A., Giltay, E. J., & Penninx, B. W. (2020). Depression profilers and immuno-metabolic dysregulation: Longitudinal results from the NESDA study. Brain, Behavior, and Immunity, 88, 174–183. 32272220 10.1016/j.bbi.2020.04.002

[ref31] Lee, C.-H., & Giuliani, F. (2019). The role of inflammation in depression and fatigue. Frontiers in Immunology, 10, 1696. 31379879 10.3389/fimmu.2019.01696 PMC6658985

[ref32] Lewis, G. F., Uffelman, K. D., Szeto, L. W., & Steiner, G. (1993). Effects of acute hyperinsulinemia on VLDL triglyceride and VLDL apoB production in normal weight and obese individuals. Diabetes, 42(6), 833–842. 8495807 10.2337/diab.42.6.833

[ref33] Liao, S.-M., Du, Q.-S., Meng, J.-Z., Pang, Z.-W., & Huang, R.-B. (2013). The multiple roles of histidine in protein interactions. Chemistry Central Journal, 7(1), 1–12. 23452343 10.1186/1752-153X-7-44 PMC3599372

[ref34] Lin, P.-Y., Huang, S.-Y., & Su, K.-P. (2010). A meta-analytic review of polyunsaturated fatty acid compositions in patients with depression. Biological Psychiatry, 68(2), 140–147. 20452573 10.1016/j.biopsych.2010.03.018

[ref35] Liu, Q., He, H., Yang, J., Feng, X., Zhao, F., & Lyu, J. (2020). Changes in the global burden of depression from 1990 to 2017: Findings from the Global Burden of Disease study. Journal of Psychiatric Research, 126, 134–140. 31439359 10.1016/j.jpsychires.2019.08.002

[ref36] Louati, K., & Berenbaum, F. (2015). Fatigue in chronic inflammation-a link to pain pathways. Arthritis Research & Therapy, 17(1), 1–10. 26435495 10.1186/s13075-015-0784-1 PMC4593220

[ref37] Masoodi, M., Kuda, O., Rossmeisl, M., Flachs, P., & Kopecky, J. (2015). Lipid signaling in adipose tissue: Connecting inflammation & metabolism. Biochimica et Biophysica Acta *(*BBA*)*-Molecular and Cell Biology of Lipids, 1851(4), 503–518. 25311170 10.1016/j.bbalip.2014.09.023

[ref38] Milaneschi, Y., Arnold, M., Kastenmüller, G., Dehkordi, S. M., Krishnan, R. R., Dunlop, B. W., … Consortium, M. D. P. M. (2022). Genomics-based identification of a potential causal role for acylcarnitine metabolism in depression. Journal of Affective Disorders, 307, 254–263. 35381295 10.1016/j.jad.2022.03.070

[ref39] Milaneschi, Y., Lamers, F., Berk, M., & Penninx, B. W. (2020). Depression heterogeneity and its biological underpinnings: Toward immunometabolic depression. Biological Psychiatry, 88(5), 369–380. 32247527 10.1016/j.biopsych.2020.01.014

[ref40] Milaneschi, Y., Simmons, W. K., van Rossum, E. F., & Penninx, B. W. (2019). Depression and obesity: Evidence of shared biological mechanisms. Molecular Psychiatry, 24(1), 18–33. 29453413 10.1038/s41380-018-0017-5

[ref41] Mittendorfer, B., Patterson, B. W., & Klein, S. (2003). Effect of sex and obesity on basal VLDL-triacylglycerol kinetics. The American Journal of Clinical Nutrition, 77(3), 573–579. 12600845 10.1093/ajcn/77.3.573

[ref42] Mittendorfer, B., Yoshino, M., Patterson, B. W., & Klein, S. (2016). VLDL triglyceride kinetics in lean, overweight, and obese men and women. The Journal of Clinical Endocrinology & Metabolism, 101(11), 4151–4160. 27588438 10.1210/jc.2016-1500 PMC5095238

[ref43] Moriarity, D., & Alloy, L.. (2020). Inflammatory phenotype of depressive and anxious symptoms: A network perspective. Biological Psychiatry, 87(9), S48.

[ref44] Moriarity, D. P., Horn, S. R., Kautz, Haslbeck, J. M., & Alloy, L. B.. (2021). How handling extreme C-reactive protein (CRP) values and regularization influences CRP and depression criteria associations in network analyses. Brain, Behavior, and Immunity, 91, 393–403. 33342465 10.1016/j.bbi.2020.10.020 PMC7753060

[ref45] Nakamura, T., Naganuma, F., Kudomi, U., Roh, S., Yanai, K., & Yoshikawa, T. (2022). Oral histidine intake improves working memory through the activation of histaminergic nervous system in mice. Biochemical and Biophysical Research Communications, 609, 141–148. 35429681 10.1016/j.bbrc.2022.04.016

[ref46] Nielsen, S., & Karpe, F. (2012). Determinants of VLDL-triglycerides production. Current Opinion in Lipidology, 23(4), 321–326. 22617755 10.1097/MOL.0b013e3283544956

[ref47] Otte, C., Gold, S. M., Penninx, B. W., Pariante, C. M., Etkin, A., Fava, M., … Schatzberg, A. F. (2016). Major depressive disorder. Nature Reviews Disease Primers, 2(1), 1–20. 10.1038/nrdp.2016.65 27629598

[ref48] Penninx, B. W., Beekman, A. T., Smit, J. H., Zitman, F. G., Nolen, W. A., Spinhoven, P., … Assendelft, W. J. (2008). The Netherlands Study of Depression and Anxiety (NESDA): Rationale, objectives and methods. International Journal of Methods in Psychiatric Research, 17(3), 121–140. 18763692 10.1002/mpr.256 PMC6878352

[ref49] Penninx, B. W., Eikelenboom, M., Giltay, E. J., van Hemert, A. M., Riese, H., Schoevers, R. A., & Beekman, A. T. (2021). Cohort profile of the longitudinal Netherlands Study of Depression and Anxiety (NESDA) on etiology, course and consequences of depressive and anxiety disorders. Journal of Affective Disorders, 287, 69–77. 33773360 10.1016/j.jad.2021.03.026

[ref50] Rodrigues, F. A. (2019). Network centrality: An introduction. In E. E. N. Macau (Ed.), A mathematical modeling approach from nonlinear dynamics to complex systems (pp. 177–196). São José dos Campos, Brazil: Springer.

[ref51] Rush, A. J., Gullion, C. M., Basco, M. R., Jarrett, R. B., & Trivedi, M. H. (1996). The inventory of depressive symptomatology (IDS): Psychometric properties. Psychological Medicine, 26(3), 477–486. 8733206 10.1017/s0033291700035558

[ref52] Sacks, F. M., & Campos, H. (2003). Low-density lipoprotein size and cardiovascular disease: A reappraisal. The Journal of Clinical Endocrinology & Metabolism, 88(10), 4525–4532. 14557416 10.1210/jc.2003-030636

[ref53] Sasahara, I., Fujimura, N., Nozawa, Y., Furuhata, Y., & Sato, H. (2015). The effect of histidine on mental fatigue and cognitive performance in subjects with high fatigue and sleep disruption scores. Physiology & Behavior, 147, 238–244. 25921948 10.1016/j.physbeh.2015.04.042

[ref54] Soininen, P., Kangas, A. J., Würtz, P., Suna, T., & Ala-Korpela, M. (2015). Quantitative serum nuclear magnetic resonance metabolomics in cardiovascular epidemiology and genetics. Circulation: Cardiovascular Genetics, 8(1), 192–206. 25691689 10.1161/CIRCGENETICS.114.000216

[ref55] Thalacker-Mercer, A. E., & Gheller, M. E. (2020). Benefits and adverse effects of histidine supplementation. The Journal of Nutrition, 150(Supplement_1), 2588S–2592S. 33000165 10.1093/jn/nxaa229

[ref56] Valente, T. W., & Fujimoto, K. (2010). Bridging: Locating critical connectors in a network. Social Networks, 32(3), 212–220. 20582157 10.1016/j.socnet.2010.03.003 PMC2889704

[ref57] Van Borkulo, C., Boschloo, L., Borsboom, D., Penninx, B. W., Waldorp, L. J., & Schoevers, R. A. (2015). Association of symptom network structure with the course of depression. JAMA Psychiatry, 72(12), 1219–1226. 26561400 10.1001/jamapsychiatry.2015.2079

[ref58] Van Borkulo, C., van Bork, R., Boschloo, L., Kossakowski, J., Tio, P., Schoevers, R., … Waldorp, L. (2022). Comparing network structures on three aspects: A permutation test. Psychological Methods. Advance online publication. 10.1037/met0000476. 35404628

[ref59] Van Diepen, J. A., Berbée, J. F., Havekes, L. M., & Rensen, P. C. (2013). Interactions between inflammation and lipid metabolism: Relevance for efficacy of anti-inflammatory drugs in the treatment of atherosclerosis. Atherosclerosis, 228(2), 306–315. 23518178 10.1016/j.atherosclerosis.2013.02.028

[ref60] Vreijling, S. R., Penninx, B. W., Bot, M., Watkins, E., Owens, M., Kohls, E., … Brouwer, I. A. (2021). Effects of dietary interventions on depressive symptom profiles: Results from the MooDFOOD depression prevention study. Psychological Medicine, 52(15), 1–10. 10.1017/S0033291721000337 PMC9772915 33823960

[ref61] Wardenaar, K. J., van Veen, T., Giltay, E. J., den Hollander-Gijsman, M., Penninx, B. W., & Zitman, F. G. (2010). The structure and dimensionality of the inventory of depressive symptomatology self report (IDS-SR) in patients with depressive disorders and healthy controls. Journal of Affective Disorders, 125(1–3), 146–154. 20074811 10.1016/j.jad.2009.12.020

[ref62] Zhang, C., Wang, K., Yang, L., Liu, R., Chu, Y., Qin, X., … Yu, H. (2018). Lipid metabolism in inflammation-related diseases. The Analyst, 143(19), 4526–4536. 30128447 10.1039/c8an01046c

[ref63] Zhong, S., Li, L., Shen, X., Li, Q., Xu, W., Wang, X., … Yin, H. (2019). An update on lipid oxidation and inflammation in cardiovascular diseases. Free Radical Biology and Medicine, 144, 266–278. 30946962 10.1016/j.freeradbiomed.2019.03.036

