# Supplementary material for: A network analysis of depressive symptoms and metabolomics
Source: Psychol Med. 2023 Apr 24;53(15):7385–94. doi: 10.1017/S0033291723001009 (PMC10719687; doi:10.1017/S0033291723001009)
Supplement: Rydin et al. supplementary material 1 — Rydin et al. supplementary material [file S0033291723001009sup001.docx]

Supplemental materials: a network analysis of depressive symptoms and metabolomics

**Journal:**

Psychological medicine

**Authors:**

Arja Rydin^[[1]](#footnote-1),^^[[2]](#footnote-2)^, Yuri Milaneschi^1,2^, Rick Quax^[[3]](#footnote-3)^, Jie Li^3^, Jos Bosch^[[4]](#footnote-4)^, Robert Schoevers^[[5]](#footnote-5)^, Erik Giltay^[[6]](#footnote-6)^, Brenda WJH Penninx^1,2,^^[[7]](#footnote-7)^, Femke Lamers^1,2^

**Correspondence address:**

Oldenaller 1
1081 HJ Amsterdam
The Netherlands

E-mail: [a.o.rydin@amsterdamumc.nl](mailto:a.o.rydin@amsterdamumc.nl)

**Number of tables and figures:**

3 figures, 2 tables

**Number of supplements:**

2 figures, 1 table

# Supplemental materials: Methods

Table S1. Variable numbers, names and grouping.

| Node number | Node name | Group | Abbreviation if applicable |
| --- | --- | --- | --- |
| 1 | Problems falling asleep | Somatic symptoms |  |
| 2 | Problems sleeping during the night |  |  |
| 3 | Early morning awakenings |  |  |
| 4 | Sleeping too much |  |  |
| 20 | Psychomotor agitation |  |  |
| 21 | Aches and pains |  |  |
| 22 | Other bodily symptoms |  |  |
| 23 | Panic/phobic symptoms |  |  |
| 24 | Constipation/diarrhea |  |  |
| 27 | Increased appetite |  |  |
| 28 | Decreased appetite |  |  |
| 29 | Increased weight |  |  |
| 30 | Decreased weight |  |  |
| 5 | Feeling sad | Mood/cogntion symptoms |  |
| 6 | Feeling irritable |  |  |
| 7 | Feeling anxious or tense |  |  |
| 8 | Mood reactivity |  |  |
| 10 | Quality of mood |  |  |
| 11 | Concentration/decision making problems |  |  |
| 12 | Self-criticism and blame |  |  |
| 13 | Future pessimism |  |  |
| 14 | Suicidal thoughts |  |  |
| 15 | Diminished interest in people/activities |  |  |
| 16 | Low energy level/fatigue |  |  |
| 17 | Diminished capacity of pleasure/enjoyment |  |  |
| 18 | Reduced interest in sex |  |  |
| 19 | Psychomotor retardation |  |  |
| 25 | Interpersonal sensitivity |  |  |
| 26 | Leaden Paralysis |  |  |
| 9 | Diurnal variation of mood | No category |  |
| 31 | Alanine (mmol/l) | Amino acids | Alanine |
| 32 | Histidine (mmol/l) |  | Histidine |
| 33 | Phenylalanine (mmol/l) |  | Phenylalanine |
| 34 | Tyrosine (mmol/l) |  | Tyrosine |
| 35 | Isoleucine (mmol/l) |  | Isoleucine |
| 36 | Leucine (mmol/l) |  | Leucine |
| 37 | Valine (mmol/l) |  | Valine |
| 38 | Apolipoprotein A-I (g/l) | Apolipoproteins | Apo A |
| 39 | Apolipoprotein B (g/l) |  | Apo B |
| 40 | Serum total cholesterol (mmol/l) | Cholesterol | Serum total cholesterol |
| 41 | Esterified cholesterol (mmol/l) |  | Esterified cholesterol |
| 42 | Free cholesterol (mmol/l) |  | Free cholesterol |
| 43 | RemNAt cholesterol (non-HDL, non-LDL -cholesterol) (mmol/l) |  | RemNAt Cholesterol |
| 44 | Total cholesterol in VLDL (mmol/l) |  | Tot chol in VLDL |
| 45 | Total cholesterol in LDL (mmol/l) |  | Tot chol in LDL |
| 46 | Total cholesterol in HDL (mmol/l) |  | Tot chol in HDL |
| 47 | Total cholesterol in HDL2 (mmol/l) |  | Tot chol in HDL2 |
| 48 | Total cholesterol in HDL3 (mmol/l) |  | Tot chol in HDL3 |
| 49 | Mean diameter for VLDL particles (nm) | Lipoprotein particle size | Mean diam VLDL |
| 50 | Mean diameter for LDL particles (nm) |  | Mean diam LDL |
| 51 | Mean diameter for HDL particles (nm) |  | Mean diam HDL |
| 52 | Serum total triglycerides (mmol/l) | Glycerides & phospholipids | Serum total triglycerides |
| 53 | Triglycerides in VLDL (mmol/l) |  | Triglycerides in VLDL |
| 54 | Triglycerides in LDL (mmol/l) |  | Triglycerides in LDL |
| 55 | Triglycerides in HDL (mmol/l) |  | Triglycerides |
| 56 | Total phosphoglycerides (mmol/l) |  | Total phosphoglycerides |
| 57 | Total cholines (mmol/l) |  | Total cholines |
| 58 | Sphingomyelins (mmol/l) |  | Sphingomyelins |
| 59 | Saturated fatty acids (mmol/l) | Fatty acids | Sat FAs |
| 60 | Monounsaturated fatty acids; 16:1, 18:1 (mmol/l) |  | Monounsat FAs |
| 61 | Polyunsaturated fatty acids (mmol/l) |  | Polyunsat FAs |
| 62 | Omega-6 fatty acids (mmol/l) |  | w6 FAs |
| 63 | Omega-3 fatty acids (mmol/l) |  | w3 FAs |
| 64 | 18:2, linoleic acid (mmol/l) |  | Linoleic acid |
| 65 | 22:6, docosahexaenoic acid (mmol/l) |  | Docosahexaenoic acid |
| 66 | Total fatty acids (mmol/l) |  | Total FAs |
| 67 | Estimated description of fatty acid chain length, not actual carbon number () | Total fatty acids and saturation measures | Est descr FA chain length |
| 68 | Estimated degree of unsaturation () |  | Est deg of unsaturation |
| 69 | Albumin (signal area) | Fluid balance | Albumin |
| 70 | Creatinine (mmol/l) |  | Creatinine |
| 71 | Citrate (mmol/l) | Glycolysis related metabolites | Citrate |
| 72 | Glucose (mmol/l) |  | Glucose |
| 73 | Lactate (mmol/l) |  | Lactate |
| 74 | Glycoprotein acetyls, mainly a1-acid glycoprotein (mmol/l) | Inflammation | Glycoprotein acetyls |
| 75 | Acetate (mmol/l) | Ketone bodies | Acetate |
| 76 | Acetoacetate (mmol/l) |  | Acetoacetate |

## Mathematical elaboration on Mixed Graphical Models

In the Methods section we briefly explained the assumptions of Mixed Graphical Models (MGM) and how these assumptions allow the dataset to contain both continuous and discrete variables. In this section we go deeper into the mathematics. For real understanding of the matter however we do refer the reader to Haslbeck (2015). For simplicity, we assume only pairwise interactions.

The dataset can be represented as a *p*-dimensional vector of random variables ***X***, with each X_s taking values in some space. We assume the graph *G* to have the Markov property over ***X***. This property means that a variable X_s is independent of its *indirect* neighbours when conditioning on X_s's *direct* neighbours. It implies that the distribution of ***X*** can be represtented as a product of so-called clique-functions. This is the case when the distribution is part of the exponential family distributions. This family consists of several types of distributions (e.g. the normal distribution, exponential distribution, binomial distribution), which can be written as a product of exponential functions.

The class of MGM considers a *p­*-dimensional random vector which has an undirected graph *G* with *p* nodes corresponding to it. We assume that the node-conditional distribution of each node X_s is given by an arbitrary univariate exponential familiy distribution conditioned on all the other variables, which as a result gives an explicit joint distribution. These assumptions are necessary because conditional distributions can become impossible to solve analytically, and the exponential family distributions prohibit this from happening.

The joint distribution takes in a number of parameters. How many numbers is dependent on the distributions of the other nodes. If all nodes in the network are continuous, MGM simplifies to a multitvariate Gaussian distribution, parameterised by *p* intercepts and *p*p* partial correlations. The estimation of MGM happens through an algorithm that first constructs the edge weights of each node to all other nodes of the network, then combines these edge weight for each pair of nodes, and then constructs the graph according to these edge weights.

# Supplemental materials: Results

## Descriptives


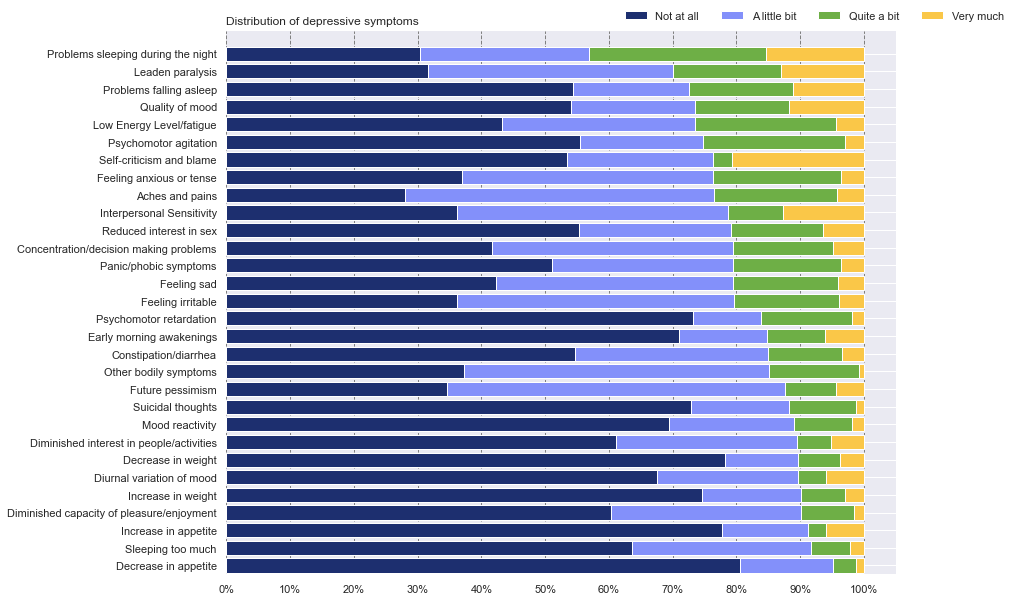


*Figure S1. Distribution of depressive symptoms at baseline (n=2498).*


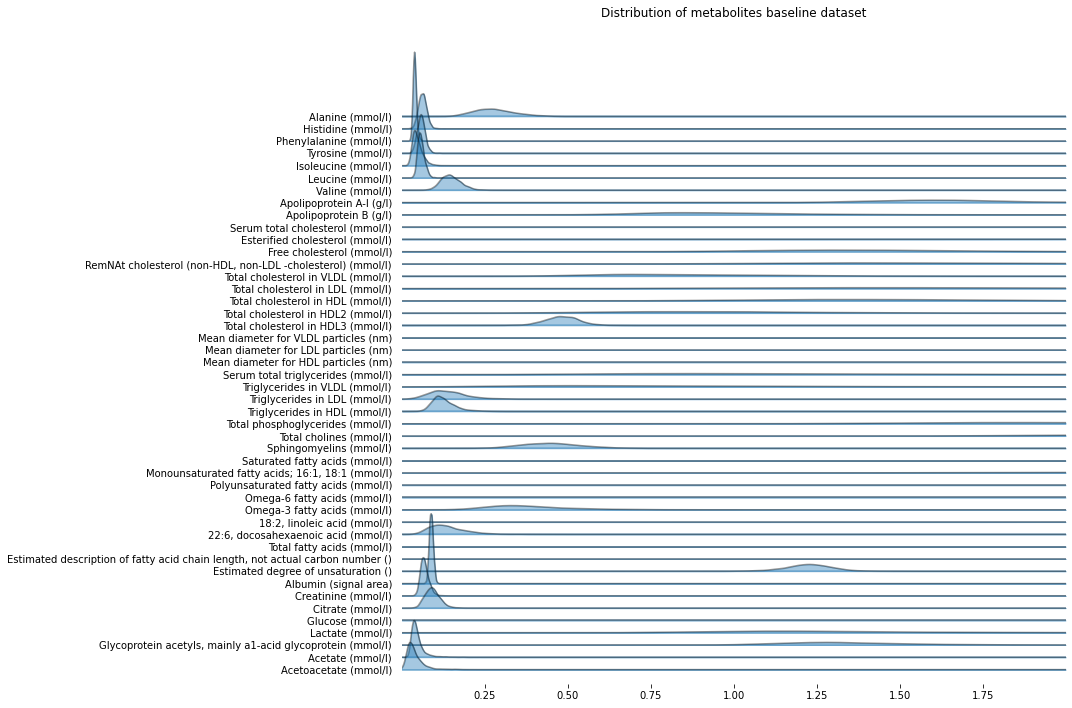


*Figure S2 Joyplot of the metabolite distribution baseline dataset (n=2498).*

1. Amsterdam UMC location Vrije Universiteit Amsterdam, Department of Psychiatry, Boelelaan 1117, Amsterdam, The Netherlands; [↑](#footnote-ref-1)
2. Amsterdam Public Health, Mental Health program, Amsterdam, The Netherlands [↑](#footnote-ref-2)
3. Computational Science Lab, Informatics Institute, Faculty of Science, University of Amsterdam, Amsterdam, The Netherlands; [↑](#footnote-ref-3)
4. Clinical Psychology, Faculty of Social and Behavioural Sciences, University of Amsterdam, Amsterdam, The Netherlands; [↑](#footnote-ref-4)
5. Department of Psychiatry, Faculty of Medical Sciences, University Medical Centre Groningen, University of Groningen, Groningen, The Netherlands; [↑](#footnote-ref-5)
6. Department of Psychiatry, Leiden University Medical Centre, Leiden University, Leiden, The Netherlands; [↑](#footnote-ref-6)
7. Department of Psychiatry and Neuroscience Campus Amsterdam, Amsterdam UMC, Vrije Universiteit, Amsterdam Public Health Research Institute, Amsterdam, The Netherlands [↑](#footnote-ref-7)
