# Supplementary figures and images for: A network analysis of depressive symptoms and metabolomics
Source: Psychol Med. 2023 Apr 24;53(15):7385–94. doi: 10.1017/S0033291723001009 (PMC10719687; doi:10.1017/S0033291723001009)

Distribution of depressive symptoms

Not at all    A little bit    Quite a bit    Very much

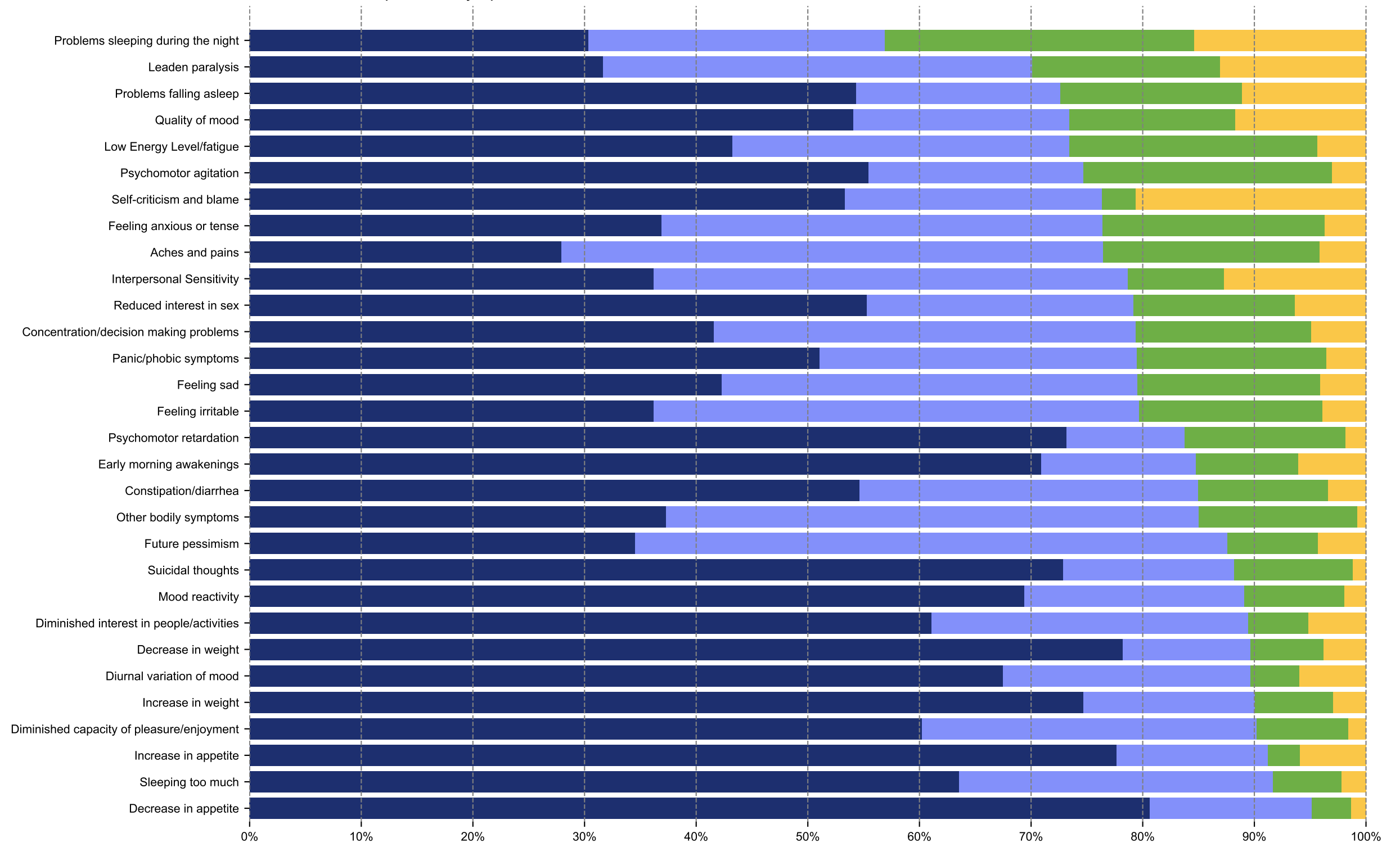

Supplement: Rydin et al. supplementary material 2 — Rydin et al. supplementary material [file S0033291723001009sup002.pdf]

Distribution of metabolites baseline dataset

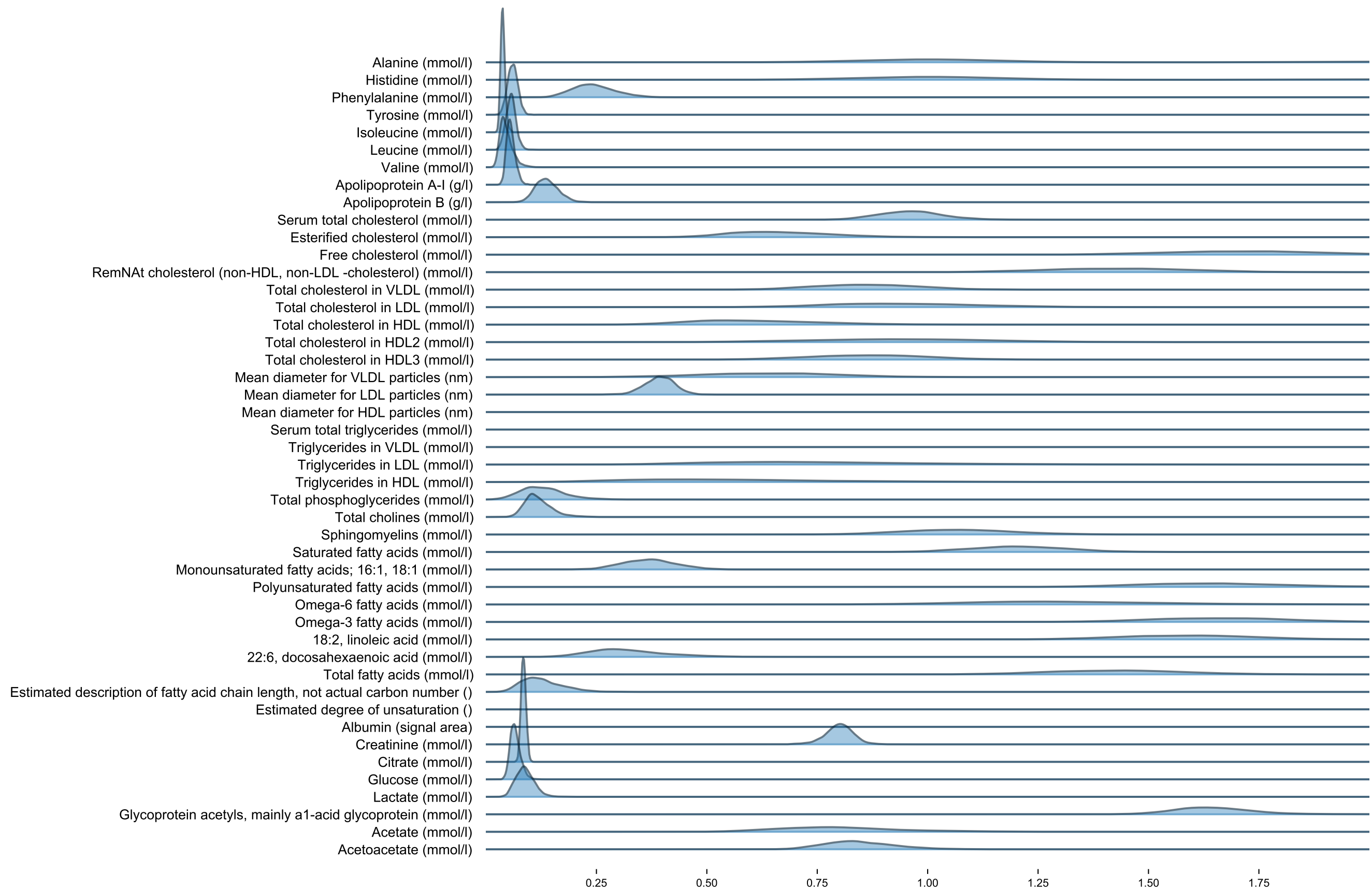

Supplement: Rydin et al. supplementary material 3 — Rydin et al. supplementary material [file S0033291723001009sup003.pdf]
